# Short-Term Outcomes of Total Knee Arthroplasty Using a Conventional, Computer-Assisted, and Robotic Technique: A Pilot Clinical Trial

**DOI:** 10.3390/jcm13113125

**Published:** 2024-05-26

**Authors:** Alexey Vladimirovich Lychagin, Andrey Anatolyevich Gritsyuk, Mikhail Pavlovich Elizarov, Yaroslav Alekseevich Rukin, Andrey Andreevich Gritsyuk, Maxim Yaroslavovich Gavlovsky, Pavel Mihailovich Elizarov, Murat Berdiyev, Eugene Borisovich Kalinsky, Ivan Antonovich Vyazankin, Nahum Rosenberg

**Affiliations:** 1 Department of Traumatology, Orthopedics, and Disaster Surgery, Federal State Autonomous Educational Institution of Higher Education, Sechenov University, Moscow 119991, Russia; dr.lychagin@mail.ru (A.V.L.); drgaamma@gmail.com (A.A.G.); elizarov_m_p@staff.sechenov.ru (M.P.E.); rukin_ya_a@staff.sechenov.ru (Y.A.R.); gritsyuk_a_@staff.sechenov.ru (A.A.G.); gavlovskiy_m_ya@student.sechenov.ru (M.Y.G.); elizarov_p_m@staff.sechenov.ru (P.M.E.); berdiev_m@student.sechenov.ru (M.B.); kalinskiy_e_b@staff.sechenov.ru (E.B.K.); vyazankin_i_a@staff.sechenov.ru (I.A.V.); 2 Specialists Center, National Insurance Institute, Haifa 3109601, Israel

**Keywords:** osteoarthritis, knee, knee arthroplasty, robotic surgery, computer-assisted surgery

## Abstract

**Background:** Total Knee Arthroplasty (TKA) is a prevalent surgical procedure for treating severe knee arthritis, aiming to alleviate pain and restore function. Recent advancements have introduced computer-assisted (CAS) and robot-assisted (RA-TKA) surgical techniques as alternatives to conventional methods, promising improved accuracy and patient outcomes. However, comprehensive comparative studies evaluating the short-term outcomes and prostheses survivorship among these techniques are limited. We hypothesized that the outcome of RA-TKA and/or CAS- TKA is advantageous in function and prosthesis survivorship compared to manually implanted prostheses. **Methods:** This prospective controlled study compared the short-term outcomes and prostheses survivorship following TKA using conventional, CAS, and RA-TKA techniques. One hundred seventy-eight patients requiring TKA were randomly assigned to one of the three surgical groups. The primary outcomes were knee function (KSS knee score) and functional recovery (KSS function score), which were assessed before surgery three years postoperatively. Secondary outcomes included prosthesis alignment, knee range of movements, and complication rates. Survivorship analysis was conducted using Kaplan–Meier curves, with revision surgery as the endpoint. **Results:** While all three groups showed significant improvements in knee function postoperatively (*p* < 0.001), the CAS and RA-TKA groups demonstrated superior prosthetic alignment and higher survivorship rates than the conventional group (100%, 97%, and 96%, respectively). However, although the RA-TKA group had a maximal 100% survivorship rate, its knee score was significantly lower than following CAS and conventional techniques (mean 91 ± 3SD vs. mean 93 ± 3SD, *p* = 0.011). **Conclusion:** The RA-TKA technique offers advantages over conventional and CAS methods regarding alignment accuracy and short-term survivorship of TKA prostheses. Since short-term prosthesis survivorship indicates the foreseen rates of mid- and long-term survivorship, the current data have a promising indication of the improved TKA prosthesis’s long-term survivorship by implementing RA-TKA. According to the presented data, although the survival rates were 100%, 97%, and 96% in the three study groups, no clinical difference in the functional outcome was found despite the better mechanical alignment and higher survivorship in the group of patients treated by the RA-TKA.

## 1. Introduction

The accuracy of implant positioning in Total Knee Arthroplasty (TKA) depends on the operative technique and the individual anatomical shape of the knee joint. The optimal placement of knee endoprosthesis determines postoperative knee function and is expressed by the implant survival rate [1,2].

The risk of the prosthesis’s aseptic loosening was reported to be increased when its mechanical axis orientation exceeded a valgus or varus deviation of 3° from the neutral coronal mechanical axis across the knee. The decrease in prosthesis survivorship becomes prominent within three years and may drop below 70% after ten years postoperatively [1]. Other studies challenged these findings when showing that moderate deviation from the optimal mechanical axis has no significant effect on the long-term survivorship of knee prostheses [2]. An additional mechanical factor contributing to prosthetic loosening is suboptimal soft tissue balancing during the surgery because the balance of the flexion–extension gaps is crucial for knee function [1]. The TKA prostheses component malposition rate, exceeding ±3° from the coronal mechanical axis, can reach 35% [3] primarily because of pathologically altered anatomical landmarks for the femoral and tibial guides [4]. Additional factors that might contribute to the risk of malposition include the surgical technique, the type of prosthesis used, and patient-specific anatomical variations. Improper alignment can cause abnormal wear patterns on the prosthetic surfaces, leading to accelerated material degradation. Thus, prosthesis malalignment, among others, can alter the knee biomechanics, leading to enhanced prosthetic wear, which may necessitate revision surgery.

Computer-assisted surgical (CAS) navigation aims to allow more accurate implant positioning according to bony landmarks, optimizing soft tissue balance and flexion–extension gaps [5,6]. Implant positioning using CAS should be more accurate than a manual technique, especially in patients with extra-articular deformity of the lower limb axis [7,8,9,10]. Previous reports have shown that in 55% of TKA by CAS, a deviation from the mechanical axis was up to 2°, which has a preferable clinical impact. It should be mentioned that TKA by CAS might encounter technical difficulties in patients with restricted hip movements due to the less effective registration ability of CAS [11].

Robot-assisted TKA (RA-TKA) is an advanced method of TKA that utilizes CAS abilities to improve the precision of implant placement. Compared to conventional guide-based TKA, RA-TKA aims to improve soft tissue protection and reduce iatrogenic bone and tissue trauma. Studies suggest that RA-TKA can improve gap balancing and component positioning, potentially enhancing surgical outcomes and reducing postoperative alignment deviations [12].

We hypothesized that the outcome of RA-TKA and/or CAS TKA is advantageous in function and prosthesis survivorship compared to manually implanted prostheses. Accordingly, the study aimed to analyze the short-term three-year survivorship of TKA surgery by CAS and RA-TKA compared to standard manual techniques. The short-term follow-up should indicate further use of RA-TKA and CAS techniques [1].

## 2. Materials and Methods

A three-arm comparative clinical study of patients who underwent primary TKA from 2019 to 2020 was conducted at the tertiary referral academic hospital. The study was conducted according to the guidelines of the Declaration of Helsinki and approved by the Institutional Review Board of Sechenov University (protocol code 25–20, date of approval 9 September 2020). All participants in the study provided their signed informed consent.

### 2.1. Study Group

Inclusion criteria: patients of both sexes, 40 to 80 years of age, with knee osteoarthritis of grade 3–4 Kellgren and Lawrence classification, i.e., with moderate and severe joint deterioration [13]. The patients with, at most, a non-life-threatening systemic disease, i.e., according to the American Society of Anesthesiologists Classification (ASA) grade ≤ 3 [14], were included.

Non-inclusion criteria: valgus/varus deformity of more than 20°; severe limitation of knee joint flexion (maximal flexion angle in the knee joint of less than 80°); severe arthritis of the ankle or hip joint; foot disorder affecting gait; patient’s refusal to participate in the study and patient’s non-compliance with the prescribed rehabilitation regimen.

Between January 2019 and September 2020, 210 patients were selected according to inclusion criteria and randomized (1:1:1) by computerized random number generation into three groups of 70 patients each for treatment by RA-TKA, manual, and CAS methods for knee prostheses implantation, as Groups A, B, and C, respectively. The operating staff was shielded from the randomization process to minimize potential bias. The patients were blinded to the allocated treatment.

### 2.2. Surgical Techniques

The preoperative evaluations included the measurement of the knee mechanical axis on the full-length standing lower limb radiographs, i.e., the angle between the mechanical axes of the femur and the tibia (hip–knee–ankle angle (HKA), normal values: 1–1.5° of varus) [15], knee joint range of motion (ROM), functional and knee KSS scores. The HKA angle is a clinical measurement used primarily to assess the alignment of the lower limb, particularly when considering TKA. This angle is formed by the intersection of two lines: one drawn from the center of the hip joint (femoral head) to the center of the knee joint (knee intercondylar notch) and the other from the center of the knee joint to the center of the ankle joint (talar dome) [16].

Following the clinical examination and preoperative planning, the patients underwent primary TKA using the following techniques:

Group A—using active-autonomous RA-TKA system (THINK Surgical TSolution One^®^, Curexo Technology, Fremont, CA, USA). The method utilizes radiographic three-dimensional registration of knee configuration with subsequential planning of precise bone cuts by a robotic arm equipped with a bone miller in the optimal configuration for the implant components implantation in the distal femur and proximal tibia [17]. This system integrates presurgical planning and intraoperative robotic execution to enhance surgical precision and outcomes in knee replacement procedures. The presurgical planning is based on computed tomography images of the patient’s knee and creating a three-dimensional virtual model. This model allows for the customization of the placement and size of the knee implant based on the patient’s unique anatomy, aiming to optimize the knee’s mechanical alignment and kinematics. During surgery, a fully robotic arm is used for the bone resections as outlined in the preoperative plan.

Group B—using a conventional set of tools for manual technique (MT) supported by intra and extramedullary guides.

Group C—using standard surgical instruments in conjunction with intra and extramedullary guides, supplemented by CAS technology (Zimmer^®^ CAS ORTHOsoft^®^ Knee 2.2 Universal computer navigation, Montreal, QC, Canada) by the digitalization of bony landmarks. This system provides real-time, precise guidance during the TKA [18]. The method utilizes an infrared camera, optical trackers, a computer workstation, and specialized software. The infrared camera captures the position of the optical trackers attached to the patient’s anatomy and surgical instruments. The computer workstation processes these data and provides real-time feedback to the surgeon through the specialized software interface. The preoperative planning involves obtaining detailed imaging studies from CT scans, which are then uploaded into the system. The software reconstructs a 3D model of the patient’s knee, allowing the surgeon to plan the optimal implant size, positioning, and alignment. This preoperative plan serves as a roadmap for intraoperative navigation. During the surgery, optical trackers are affixed to the patient’s femur and tibia using percutaneous pins. The infrared camera captures the position of these trackers, which is essential for accurate navigation. The surgeon uses a digitizing probe to map the anatomical landmarks and the bone surface of the femur and tibia. This information is integrated into the 3D model, ensuring the system accurately represents the patient’s anatomy. The computer navigation system guides the surgeon in making precise bone cuts. Real-time feedback on the position and alignment of the cutting guides helps ensure that the bone resections are accurate. The system continually monitors and displays the mechanical axis alignment, which is crucial for achieving optimal postoperative outcomes. The surgeon can adjust the implant placement to achieve the desired alignment and balance by referencing the preoperative plan and real-time intraoperative data. After the implants are placed, the system allows for verification of the alignment and positioning. This step is crucial to ensure the implants are correctly oriented and restore the knee’s mechanical axis.

Three experienced surgeons performed the surgical procedures in all groups in equal proportions. All the surgeries were under spinal anesthesia with intravenous sedation and standard intraoperative monitoring.

A standard medial parapatellar approach with patella eversion selectively accompanied by patellar resurfacing based on the degree of cartilage wear, removal of osteophytes, and circular denervation was performed. No tourniquet was used, and hemostasis during surgery was performed with electrocoagulation. Zimmer^®^ Persona knee implants with posterior cruciate ligament (CR) preservation were inserted using cement fixation and a fixed-bearing insert (Zimmer, Inc., Warsaw, IN, USA).

Mechanical alignment of the lower limb axis was targeted for HKA below 2°.

Postoperatively, all patients in the three groups received systemic multimodal analgesia and standard mechanical and pharmacologic thromboprophylaxis.

All patients, within the first hours after surgery, flexed and extended the operated knee, initially passively (up to 6 h), then actively, under the control of the rehabilitation therapists, who were not informed about dividing patients into groups. The patient was mobilized and allowed to ambulate from the first day after surgery. All the patients received a standard rehabilitation protocol with limited load bearing on the operated lower limb for up to three weeks.

### 2.3. Postoperative Follow-Up

Then, follow-up examinations were carried out one year, two years, and three years after the surgery by the Knee Society Clinical Rating System (KSS) [19]. The KSS reflects local objective knee-related abilities (knee score, 0–100 points scale, reflects pain, range of motion, and knee joint stability) and general functional abilities related to the knee (functional score, 0–100 points scale, reflects walking distance, ability to climb stairs, etc.). To reduce the bias effects, the research assistant who recorded the scoring data was blinded to the division into the study groups.

The range of motion in the knee joint was measured in the supine position using a hand-held goniometer. Weight-bearing knee radiography in two projections (long-leg radiograph in anteroposterior and sagittal projections) was performed before, after surgery, and at the follow-up. After surgery, the mechanical axis angle (HKA) was compared to the preoperative values. Deviation of the HKA of less than ±1° was considered an “excellent” result, ±1° to ±3° was considered a “good” result, and greater than ±3° was considered a “marginal” outcome [15].

### 2.4. Statistical Analysis

The one-way ANOVA compared the clinical data for parametric distribution; otherwise, the Kruskal–Wallis test was implemented. The comparison of preoperative and postoperative parameters in patients was performed by paired t-test in parametric distribution; otherwise, the Wilcoxon Signed Rank Test was used (by IBM SPSS Statistics 22.0 package, IBM Corp. (2013) IBM SPSS Statistics for Windows, Version 22.0. IBM Corp., Armonk, NY, USA). The level of significance was set at *p* < 0.05.

The sample size for each study group (60 patients per group) was calculated based on detecting a minimum difference of means of 10%, assuming an expected standard deviation of 15%, a desired test power of 90%, and a significance level (α) of 0.05. (calculated by SIGMASTAT software, version 2, SPSS Inc., Chicago, IL, USA). A threshold of 10% difference in the KSS scores has been determined previously as the minimal clinically important difference (MCID) after TKA [20].

Kapan–Meier survival analysis [21] determined the implant survival rate. Revision surgery due to septic and aseptic loosening was considered the endpoint criterion for prosthetic failure.

The methodology employed in this study adhered to the principles outlined in the Consolidated Standards of Reporting Trials (CONSORT) 2010 guidelines. The CONSORT checklist can be found in Appendix A (Table A1).

## 3. Results

**Figure 1:**
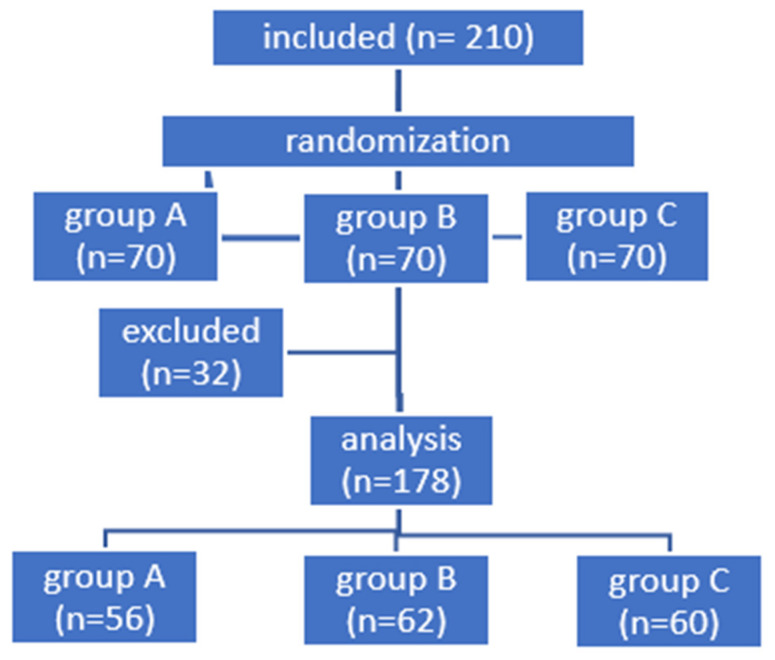
Randomization scheme of patients into study groups.

During three years of follow-up, 32 patients, 15% of the study group, were excluded (14 in Group A, 8 in Group B, 10 in Group C) for the following reasons: 10 patients refused further participation in the study, 22 patients missed follow-up examinations (Figure 1).

Among the 178 patients, 134 were women (75.2%) and 44 men (24.8%), with a mean age of 68 ± 11.3 (SD) years for men and 66 ± 12.5 (SD) years for women (min 39 years, max 80 years, *p* = 0.262). Median Body Mass Index was 33.6 kg/m^2^ (min 25.1 kg/m^2^, max 34.7 kg/m^2^, *p* = 0.218, Table 1).

Following the surgeries, a significant reduction in the HKA was achieved (*p* < 0.001, *p* = 0.001, *p* = 0.002 for Groups A, B, and C, respectively, Wilcoxon Signed Rank Test), bringing the average values into the desired range (Table 2). In Group A, postoperative deviation of the HKA of less than 1° was observed in 72% of cases, from 1° to 3° in 28%, and no deviation of more than 3°. In Group B, a deviation of less than 1° occurred in 30% of cases, from 1° to 3°—55%, and more than 3° in 15% of cases. In the patients of Group C, the deviation up to 1° occurred in 45% of patients, from 1° to 3°—48%, and more than 3°—7%.

Before surgery, all three groups had no significant difference in knee and functional scores (29 ± 4 SD, 31 ± 6 SD; 28 ± 4 SD, 34 ± 6 SD; 29 ± 4 SD, 36 ± 7 SD of KSS and functional scores for Groups A, B, and C, respectively, *p* > 0.05, Kruskal–Wallis One-Way Analysis of Variance on Ranks test).

Following three years of follow-up, all the patients had full extension of the operated knee. There was no significant difference in knee flexion among the three tested groups (118° ± 15° SD, 108° ± 10° SD, 112° ± 12° SD in Groups A, B, and C, respectively, *p* > 0.05, Kruskal–Wallis One-Way Analysis of Variance on Ranks followed by Pairwise Multiple Comparison Procedures—Dunn’s Method).

**Figure 2:**
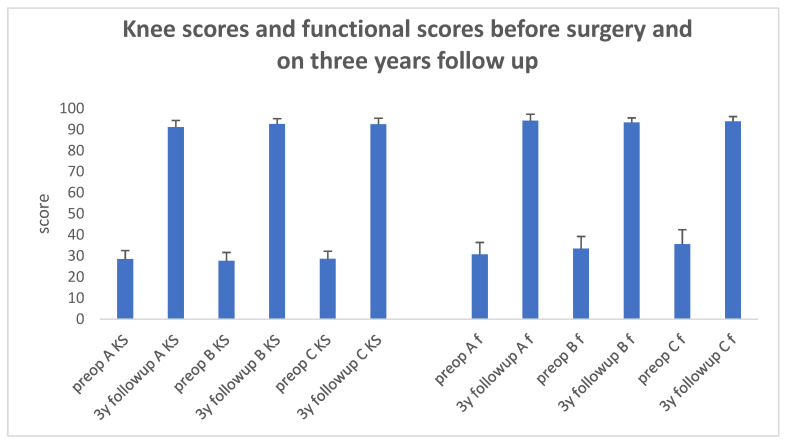
Knee scores and functional scores before surgery and on three years follow-up. Mean values of knee and functional scores are presented. Vertical bars represent the standard deviation from the mean. In all study groups, a significant increase in values on the three-year follow-up compared to the preoperative scores is apparent (*p* < 0.001, Kruskal–Wallis One-Way Analysis of Variance on Ranks followed by Pairwise Multiple Comparison Procedures—Dunn’s Method). KS—knee score. f—functional score. A, B, and C—represent Groups A, B, and C, respectively.

On the three-year follow-up, all three groups showed a significant improvement in both KSS knee and functional scores in comparison to their preoperative values (*p* < 0.001, Kruskal–Wallis One-Way Analysis of Variance on Ranks followed by Pairwise Multiple Comparison Procedures—Dunn’s Method, Figure 2).

On the three-year follow-up evaluation, there was no significant difference in knee scores between Groups B and C (93 ± 3 SD in both groups), but the scores of Groups B and C were significantly higher than in Group A (91 ± 3 SD; *p* = 0.011, one-way ANOVA, followed by Tukey’s post hoc test).

During the three-year follow-up evaluation, there was no significant difference in knee functional scores among all three study groups (*p* = 0.245, one-way ANOVA).

**Figure 3:**
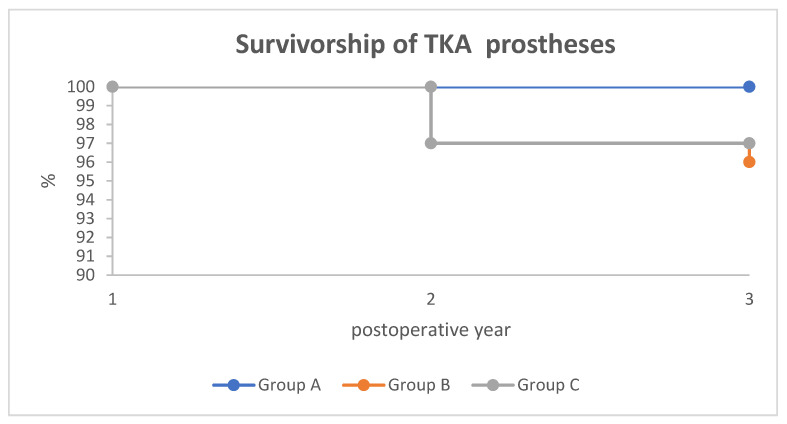
Survivorship analysis (according to the Kaplan–Meier method, failure is considered as prosthesis revision surgery) of implanted knee prostheses in all study groups. At the three years follow-up, the survival rates were 100%, 97%, and 96% 1 in Groups A, C, and B, respectively.

No complications were reported in Group A. In Group B, one patient (1.6%) was diagnosed with aseptic instability of the endoprosthesis components one year after the operation, which required revision intervention. Another patient (1.6%) was diagnosed with deep prosthetic joint infection three years after the operation and underwent two-stage surgical treatment. In Group C, aseptic loosening of the prosthesis was diagnosed in two (3.3%) patients three years after the operation, which required revision intervention. Thus, the survivorship rate of the three years of prostheses was 100%, 97%, and 96% in Groups A, C, and B, respectively (Figure 3).

## 4. Discussion

There were no statistically significant personal and demographic differences between the patients in the three study groups (sex, age, BMI, operated side). The study groups also had statistically similar preoperative knee and functional scores. Therefore, the randomization of the patients created comparable groups for further postoperative comparison.

The accuracy of positioning the implant components in TKA is a major part of the surgical procedure that should affect the implant’s survival and the treatment’s functional results. RA-TKA and CAS methods aim to correct the mechanical alignment axis of the lower limb to improve the function and survivorship of knee prostheses.

RA-TKA and CAS are advanced techniques employed to enhance the precision of implant positioning and alignment during TKA. Their primary goal is to correct the mechanical alignment axis of the lower limb, which is crucial for optimizing knee function and prolonging the survivorship of the knee prostheses [22,23].

Mechanical alignment plays a critical role in the success of TKA because malalignment is a major contributor to implant failure and adverse patient outcomes. Conventional TKA relies on the surgeon’s experience and manual techniques to achieve proper alignment, which can be variable. In contrast, RA-TKA and CAS provide real-time feedback and guidance, improving the accuracy and consistency of bone cuts and implant positioning. RA-TKA and CAS can achieve alignment within 3 degrees of the intended target in over 90% of cases, compared to 70–80% with conventional techniques [23,24].

Previously published meta-analysis data showed that RA-TKA and CAS significantly improve mechanical axis alignment [24]. This improved alignment aims to achieve more natural knee kinematics and reduced wear on the prosthetic components, which are critical factors in enhancing the function and extending the survivorship of knee prostheses.

The extent of the correlation between alignment accuracy and improved functional outcomes in TKA remains controversial. Although it is intuitively believed that perfect alignment of the TKA prosthesis should lead to more balanced loading across the knee joint during motion, thereby minimizing uneven wear and stress on implant surfaces and reducing the risk of aseptic loosening with enhanced functional outcomes, this notion has been previously challenged [25]). In the present report, we contribute to this controversy by demonstrating that while better alignment of prostheses achieved through RA-TKA resulted in a perfect 100% short-term survivorship, it was associated with less favorable KSS scores than manual implantation. This issue warrants further investigation through long-term survivorship studies.

A key advantage of RA-TKA and CAS precision is the potential for less invasive procedures, leading to quicker recovery and reduced postoperative pain. These technologies provide surgeons with detailed 3D models of the patient’s anatomy and real-time tracking of surgical instruments, enabling them to execute the surgical plan with high fidelity [26]. While RA-TKA and CAS offer considerable advantages in achieving optimal mechanical alignment, it is essential to note that the technology requires significant training and familiarity.

Therefore, RA-TKA and CAS are transformative approaches in knee arthroplasty that enhance the precision of mechanical alignment corrections. This precision is fundamental in improving knee prostheses’ functional outcomes and longevity. The consistent replication of intended alignment parameters across different patient anatomies improves the postoperative outcomes. It aims to contribute to the prosthetic knee’s long-term survivorship by minimizing wear and potential failures, thereby enhancing overall patient quality of life. In particular, it has been previously shown that RA-TKA results in better precision in achieving planned limb alignment and implant positioning than conventional methods [27]. This precision aims to improve functional outcomes for patients through more physiological joint stability and mobility.

A significant advantage of the RA-TKA method is the potential for reduced recovery time. The precise cutting minimizes damage to surrounding tissues, leading to faster recovery and less postoperative pain for patients. Additionally, the accuracy provided by the robotic system may reduce the need for subsequent corrective surgeries, thereby improving overall patient satisfaction and reducing healthcare costs.

The present study contributes to these different opinions on the efficiency of RA-TKA and CAS. The analysis of mechanical axis correction outcomes reveals that Group A attained an “excellent” outcome 2.3 times more often than Group B and 1.5 times more than Group C. Additionally, the occurrence of a “good” outcome in Group A was 1.6 times greater than in Group B and 1.4 times greater than in Group C. These data underscored the superiority of RA-TKA in achieving more accurate implant placement compared to conventional manual techniques, with or without CAS navigation, corroborating findings from previous research [14]. This accuracy is linked to a higher implant survival rate observed over a three-year follow-up period. This implies that precise correction of the knee’s mechanical axis contributes to the enhanced longevity of the prosthesis. However, this improvement in mechanical accuracy does not necessarily translate to better functional outcomes compared to the other two techniques; the functional scores were similar across all groups, with the RA-TKA group even displaying a marginally lower knee score than the CAS and manual technique groups. Hence, it is inferred that RA-TKA may reduce prosthetic loosening, as evidenced by the highest prosthetic survival rate. Yet, it does not significantly impact knee functionality relative to other methods. Similar to the previous report [26], this observation is further validated by the performance of CAS-assisted prosthesis implantation, which offered greater precision than manual methods but was less precise than RA-TKA, resulting in an intermediate three-year survival rate (Figure 3).

A notable limitation of this study is its reliance on short-term follow-up data to formulate conclusions. It is important to recognize that, according to current research, manually implanted TKA demonstrated long-term survivorship (spanning ten to fifteen years) exceeding 90%, particularly when prosthetic failure is attributed to loosening [27,28,29]. These studies indicate a commencement of decline in prosthesis survivorship within the short-term follow-up period (3–5 years post-surgery). Consequently, addressing the less than 10% gap of desired improvement in survivorship rates is crucial for enhancing expected long-term outcomes. Therefore, the significance of short-term survivorship data cannot be understated, as it plays a critical role in the selection process for surgical techniques going forward. Our findings suggest that RA-TKA exhibits the potential to boost the long-term survivorship of prostheses based on the short-term data observed. Further long-term survivorship studies following this pilot clinical trial should give a substantial answer to this current uncertainty. According to the presented promising data, further midterm and long-term follow-ups will be performed to support this hypothesis.

## 5. Clinical Relevance

Total knee arthroplasty (TKA) is a crucial surgical procedure for treating severe knee arthritis, aiming to alleviate pain and improve joint function. The evolution of TKA includes the development of CAS and RA-TKA techniques, which are believed to enhance surgical precision and patient outcomes compared to traditional methods. Despite these advancements, there is a lack of rigorous comparative research, especially regarding the longevity of implants among these methods [30].

This study evaluated and compared the short-term outcomes and implant survival rates following TKA using conventional, CAS, and RA-TKA methods in a prospective, controlled setting. Outcomes measured included improved knee function, assessed by the Knee Society Score (KSS) knee score, and functional recovery, gauged by the KSS function score, evaluated up to three years post-surgery. The early results showed significant improvements in knee function across all groups. Interestingly, while the RA-TKA group achieved a perfect survivorship rate of 100%, its functional knee-related scores (KSS scores) were slightly lower than those seen with CAS and conventional methods.

The RA-TKA approach thus demonstrates potential advantages in terms of alignment accuracy and short-term prosthetic survival, suggesting promising implications for the longevity of implants in TKA procedures. This study underscores the need for continued research to validate these findings and explore the long-term outcomes of different TKA techniques, informing surgical practice and improving patient care.

## 6. Conclusion

The results indicate that the prostheses’ 100% short-term survival following RA-TKA may improve long-term survival, potentially enhancing the implants’ biomechanics. These short-term survivorship data support the notion that RA-TKA could improve the longevity of TKA implants over a prolonged duration. Overall, the results demonstrated that in the short-term, computer-assisted techniques did not lead to clinically worse outcomes.

Despite the group treated with RA-TKA achieving better mechanical alignment, no clinical difference in functional outcomes was observed among the three study groups.

## Figures and Tables

**Table 1:** Study group characteristics. Mean value ± standard deviation is presented.

Demographic Parameters		Group A (N = 56)	Group B (N = 62)	Group C (N = 60)	*p* *
Sex	Women/men	44/12	47/15	43/17	0.240
Age		67.7 ± 10.2	66.5 ± 8.7	65.7 ± 9.1	0.262
Body mass index (BMI)		31.4 ± 2.6	32.1 ± 4.1	31.2 ± 3.8	0.218
Side	R/L	30/26	32/30	33/27	0.720

* one-way ANOVA.

**Table 2:** The lower limb’s mechanical axis (HKA) before and after surgery. Mean values ± standard deviation are presented.

HKA (Degrees of Varus)	Group A (N = 56)	Group B (N = 62)	Group C (N = 60)	*p* * Pre-op vs. Post-op *
Pre-op	9.21 ± 1.95	10.58 ± 1.92	10.07 ± 2.15	0.001
Post-op	0.38 ± 0.49	2.25 ± 1.08	0.94 ± 0.63	

* Wilcoxon Signed Rank Test.

## Data Availability

Data supporting reported results are kept at Department of Traumatology, Orthopedics, and Disaster Surgery, Federal State Autonomous Educational Institution of Higher Education, Sechenov University, Moscow, Russia.

## Appendix A. Short-Term Outcomes of Total Knee Arthroplasty Using a Conventional, Computer-Assisted, and Robotic Technique: A Pilot Clinical Trial

**Table A1:** CONSORT 2010 checklist of information to include when reporting a randomized trial [31]. + indicates the addressed subject (p—page number in the text).

Section/Topic	Item No	Checklist Item	Reported on Page No
Title and abstract			
	1a	Identification as a randomized trial in the title	The title is according to the editor’s suggestion p1
	1b	Structured summary of trial design, methods, results, and conclusions	+ p1
Introduction			
Background and objectives	2a	Scientific background and explanation of rationale	+ p1–2
	2b	Specific objectives or hypotheses	+ p2
Methods			
Trial design	3a	Description of trial design (such as parallel, factorial) including allocation ratio	+ p2–3
	3b	Important changes to methods after trial commencement (such as eligibility criteria), with reasons	+ p5
Participants	4a	Eligibility criteria for participants	+ p2
	4b	Settings and locations where the data were collected	+ p2
Interventions	5	The interventions for each group with sufficient details to allow replication, including how and when they were actually administered	+ p3–4
Outcomes	6a	Completely defined pre-specified primary and secondary outcome measures, including how and when they were assessed	+ p4–5
	6b	Any changes to trial outcomes after the trial commenced, with reasons	No changes p5
Sample size	7a	How sample size was determined	+ p4
	7b	When applicable, explanation of any interim analyses and stopping guidelines	Not applicable
Randomization			
Sequence generation	8a	Method used to generate the random allocation sequence	+ p2
	8b	Type of randomization; details of any restriction (such as blocking and block size)	+ p2–3
Allocation concealment mechanism	9	Mechanism used to implement the random allocation sequence (such as sequentially numbered containers), describing any steps taken to conceal the sequence until interventions were assigned	+ p2
Implementation	10	Who generated the random allocation sequence, who enrolled participants, and who assigned participants to interventions	+ p2
Blinding	11a	If done, who was blinded after assignment to interventions (for example, participants, care providers, those assessing outcomes) and how	+ p3
	11b	If relevant, description of the similarity of interventions	Not relevent
Statistical methods	12a	Statistical methods used to compare groups for primary and secondary outcomes	+ p4–5
	12b	Methods for additional analyses, such as subgroup analyses and adjusted analyses	+ p4–5
Results			
Participant flow (a diagram is strongly recommended)	13a	For each group, the numbers of participants who were randomly assigned, received intended treatment, and were analyzed for the primary outcome	+ p5
	13b	For each group, losses and exclusions after randomization, together with reasons	+ p5
Recruitment	14a	Dates defining the periods of recruitment and follow-up	+ p2
	14b	Why the trial ended or was stopped	+ Short term p1
Baseline data	15	A table showing baseline demographic and clinical characteristics for each group	+ p2
Numbers analyzed	16	For each group, number of participants (denominator) included in each analysis and whether the analysis was by original assigned groups	+p5
Outcomes and estimation	17a	For each primary and secondary outcome, results for each group, and the estimated effect size and its precision (such as 95% confidence interval)	+ p6–8
	17b	For binary outcomes, presentation of both absolute and relative effect sizes is recommended	No binary outcomes
Ancillary analyses	18	Results of any other analyses performed, including subgroup analyses and adjusted analyses, distinguishing pre-specified from exploratory	+ Survivorship analysis p5
Harms	19	All important harms or unintended effects in each group	+ Complications p7
Discussion			
Limitations	20	Trial limitations, addressing sources of potential bias, imprecision, and, if relevant, multiplicity of analyses	+ p3–4
Generalisability	21	Generalisability (external validity, applicability) of the trial findings	+ p10
Interpretation	22	Interpretation consistent with results, balancing benefits and harms, and considering other relevant evidence	+ p10–11
Other information			
Registration	23	Registration number and name of trial registry	+ p2
Protocol	24	Where the full trial protocol can be accessed, if available	In the IM Sechenov University
Funding	25	Sources of funding and other support (such as supply of drugs), role of funders	No external funding
